# Water permeation through the internal water pathway in activated GPCR rhodopsin

**DOI:** 10.1371/journal.pone.0176876

**Published:** 2017-05-11

**Authors:** Katsufumi Tomobe, Eiji Yamamoto, Kholmirzo Kholmurodov, Kenji Yasuoka

**Affiliations:** 1 Department of Mechanical Engineering, Keio University, 3-14-1 Hiyoshi, Kohoku-ku, Yokohama 223-8522, Japan; 2 Graduate School of Science and Technology, Keio University, 3-14-1 Hiyoshi, Kohoku-ku, Yokohama 223-8522, Japan; 3 Frank Laboratory of Neutron Physics, Joint Institute for Nuclear Research, Dubna, 141980, Russia; 4 Dubna State University, Dubna, 141980, Russia; University of Lincoln, UNITED KINGDOM

## Abstract

Rhodopsin is a light-driven G-protein-coupled receptor that mediates signal transduction in eyes. Internal water molecules mediate activation of the receptor in a rhodopsin cascade reaction and contribute to conformational stability of the receptor. However, it remains unclear how internal water molecules exchange between the bulk and protein inside, in particular through a putative solvent pore on the cytoplasmic. Using all-atom molecular dynamics simulations, we identified the solvent pore on cytoplasmic side in both the Meta II state and the Opsin. On the other hand, the solvent pore does not exist in the dark-adapted rhodopsin. We revealed two characteristic narrow regions located within the solvent pore in the Meta II state. The narrow regions distinguish bulk and the internal hydration sites, one of which is adjacent to the conserved structural motif “NPxxY”. Water molecules in the solvent pore diffuse by pushing or sometimes jumping a preceding water molecule due to the geometry of the solvent pore. These findings revealed a total water flux between the bulk and the protein inside in the Meta II state, and suggested that these pathways provide water molecules to the crucial sites of the activated rhodopsin.

## Introduction

**Fig 1:**
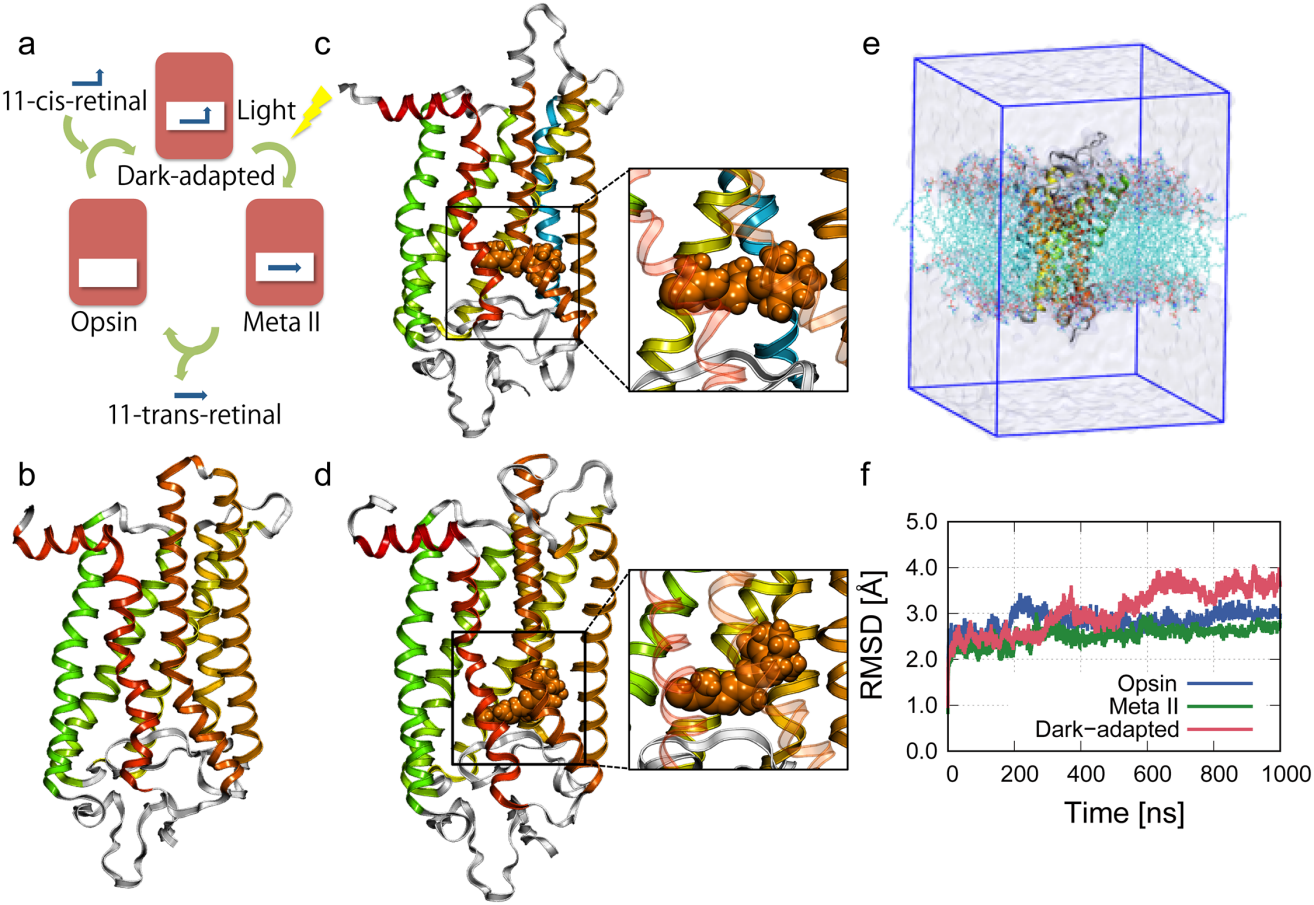
Crystal structure of three states of rhodopsin and stability of our systems. (a) Activation cycle of rhodopsin. Through several intermediate states including the Meta II state, the rhodopsin is decayed into the Opsin and the 11-trans-retinal. Crystal structures of the rhodopsin in (b) the Opsin, (c) the Meta II state, and (d) the dark-adapted rhodopsin are shown. The retinal is shown in orange VDW format. (e) The system used for MD simulations of the dark-adapted rhodopsin in a POPC lipid bilayer. The rhodopsin and lipids are shown in cartoon and cyan lines, respectively. Explicit water molecules correspond to the upper and lower transparent coatings. The blue line of the box is a periodic boundary. (f) Backbone root mean square deviations (RMSDs) of the dark-adapted rhodopsin, the Meta II state and the Opsin. We calculated the RMSDs only using helical parts of rhodopsin.

G-protein-coupled receptors (GPCRs) are transmembrane (TM) proteins that transmit a signal from the extracellular to the cytoplasmic side of cell membranes via G-proteins. GPCRs have become the targets of 30% of marketed drugs and are still attractive materials for pharmaceutical and biophysical studies [1–3]. A light-driven GPCR protein, called rhodopsin, is composed of three parts: seven TM helixes, a small helix parallel to the membrane that works as an anchor for the interaction with the G-protein, and a light-sensitive chromophore (11-cis retinal) [4]. When the dark-adapted rhodopsin absorbs light, 11-cis-retinal is isomerized to 11-trans-retinal within 200 fs, which is one of the fastest chemical reactions in the human body [5–7]. This reaction triggers cascade reactions in the rhodopsin. Through several intermediate states including the Meta II state [4, 8, 9], a Schiff base linkage between the 11-trans-retinal and K296 is disrupted by hydrolysis (see Fig 1a–1d). Finally, the rhodopsin decays into the active state of the rhodopsin, called Opsin, and the 11-trans-retinal [10].

Rhodopsin has two pores in the cell membrane, called ligand pores. One is located between TM5 and TM6, and the other is located between TM1 and TM7, and two ligand pores function in release and uptake of the retinal [11–13]. In addition to the two pores, it is thought that the Meta II state and Opsin have one narrow pore on the cytoplasmic side [14, 15]. This narrow pore, called a ‘solvent pore’, was suggested by Angel *et al.* using radiolytic hydroxyl labeling and liquid chromatography coupled to mass spectroscopy [16]. The solvent pore is thought to provide water molecules from the cytoplasmic bulk to the retinal-binding pocket. Because it is narrow, it only allows permeations of water molecules and some small compounds [17, 18]. The solvent pore is regarded as functionally important pathway for the cascade reaction because hydrolysis of the Schiff base linkage requires a water molecule [13]. Moreover, the internal water molecules around the retinal stabilize local electric fields by hydrating near polar and charged amino acids [19]. Other GPCRs also have a continuous solvent pore, and its function depends on the state of the GPCRs [20]. Additionally, water molecules passing through the solvent pore might affect important internal water molecules within the rhodopsin. In general, internal water molecules play important roles in biological materials, e.g., transferring oxygens and protons [21, 22], regulating other material permeations [23, 24], and stabilizing biological materials, such as proteins and DNA [25]. In case of the rhodopsin, internal water molecules contribute to the thermodynamic and conformational stabilities of the protein [26]. Furthermore, these water molecules contribute to protein plasticity, and they mediate the activation of protein in other GPCRs [27]. Fourier-transform infrared spectroscopy has shown that configuration of the internal water molecules changes during photoactivation process [19], which indicates that water molecules exchange between the bulk and the internal hydration sites. Although the importance of internal water molecules is well known, the precise pathways of water molecules between the bulk and protein inside remain poorly understood. Additionally, it remains difficult to investigate the dynamics of internal water molecules within the solvent pore directly by spectroscopy.

Here, using all-atom molecular dynamics (MD) simulations, we reveal details of water flux between the bulk and rhodopsin inside. Due to the different secondary and hydration structures for each intermediate in rhodopsin, we separately prepare and investigate three states of rhodopsin: the dark-adapted rhodopsin, the intermediate Meta II state, and the Opsin. Our simulations and computational analyses show that a putative solvent pore exists on the cytoplasmic side in the Meta II state and the Opsin while the solvent pore does not exist in the dark-adapted rhodopsin. We clearly provide a detailed configuration of the solvent pore, showing that there are two narrow regions that distinguish the bulk and internal hydration sites.

## Materials and methods

We used the crystal structures of bovine rhodopsin because only bovine rhodopsin can be obtained as crystal structure in the three states. The structure of dark-adapted rhodopsin (PDB ID: 1U19) [28] was used as the initial structure. The dark-adapted rhodopsin was embedded in a lipid bilayer composed of 170 POPC lipids, and these systems were hydrated in 6,499 water molecules. In the case of the Opsin, we used the Opsin structure (PDB ID: 3CAP) [29] in 166 POPC lipids and 6627 water molecules. The crystal structure of the Meta II state was obtained from PDB ID: 3PXO [30]. The system was hydrated with 7,400 water molecules with 169 POPC lipids. The Asp83, Glu122 and Glu113 were protonated in Meta II state, and Glu113 was protonated in the Opsin. Co-crystalized water molecules were utilized as initial internal water molecules while palmitic acids were removed. In all simulations, we added 5 chloride, 2 chloride, and 1 sodium ions to neutralize the system of the dark-adapted rhodopsin, the Meta II state, and the Opsin, respectively. These ions were randomly placed in bulk of the systems. A disulfide bond was added between Cys110 and Cys187. All MD simulations were conducted using NAMD 2.9 [31] software. The CHARMM 36 forcefield was used for the lipids [32] and the protein [33] and TIP3P model modified for the CHARMM forcefield [34]. Force field parameters for the ligand molecules were generated with the CHARMM General Force Field [35]. Using the VMD [36], we built the initial structure of our systems. First, we embedded the rhodopsin on the center of membrane bilayer, and removed overlapping lipid molecules and overlapping water molecules. Snapshot of the system is shown in Fig 1e. After the 1 *μ*s simulations, box sizes of the simulation systems were 80 × 80 × 116, 81 × 81 × 113, and 81 × 81 × 116 Å^3^ for the systems of the dark-adapted rhodopsin, the Meta II state, and the Opsin, respectively.

After the initial system setup, the system was subject to 2000 steps minimization using conjugate gradient and line search method, and *NPT* simulations of 5 ns were performed with the constraint on the protein to initial positions by a harmonic potential. Langevin dynamics thermostat and Nosé-Hoover Langevin piston barostat were applied for temperature and pressure coupling at 310 K and 1 bar. Van der Waals interactions were smoothly truncated by force switching from 10 to 12 Å. A time step of 2 fs was used with the SHAKE algorithm applied to constrain the bond lengths involving hydrogen atoms. All systems were subjected to periodic boundary conditions. Electrostatic interactions were computed using the particle-mesh Ewald method. Each simulation was performed for 1 *μ*s and the analysis were conducted over the last 0.8 *μ*s trajectories. The data for the first 0.2 *μ*s were discarded for the equilibration of the simulation, which was judged by backbone root mean square deviation (RMSD) (see Fig 1f).

To validate the reproducibility of our results, we performed the same systems using AMBERff99SB-ildn [37] for protein, Slipid for POPC [38], and TIP3P water models [39]. Additional calculations were performed with Gromacs 5.1.2 [40]. The systems were subject to pressure scaling to 1 bar using Parrinello-Rahman barostat [41], temperature scaling to 310 K using velocity-rescaling method [42]. Force field parameters for the protonated 11-cis-retinal in the rhodopsin and the deprotonated all-trans-retinal in Meta II state were prepared using antechamber with the General Amber Force Field [43] and the AMI-BCC point charges. 1 ns NVT and NPT simulations were performed with the restrained protein for equilibration. NPT simulations were performed for 1 *μ*s with 2.0 fs time-step.

## Results

### Accessibility of water molecules

**Fig 2:**
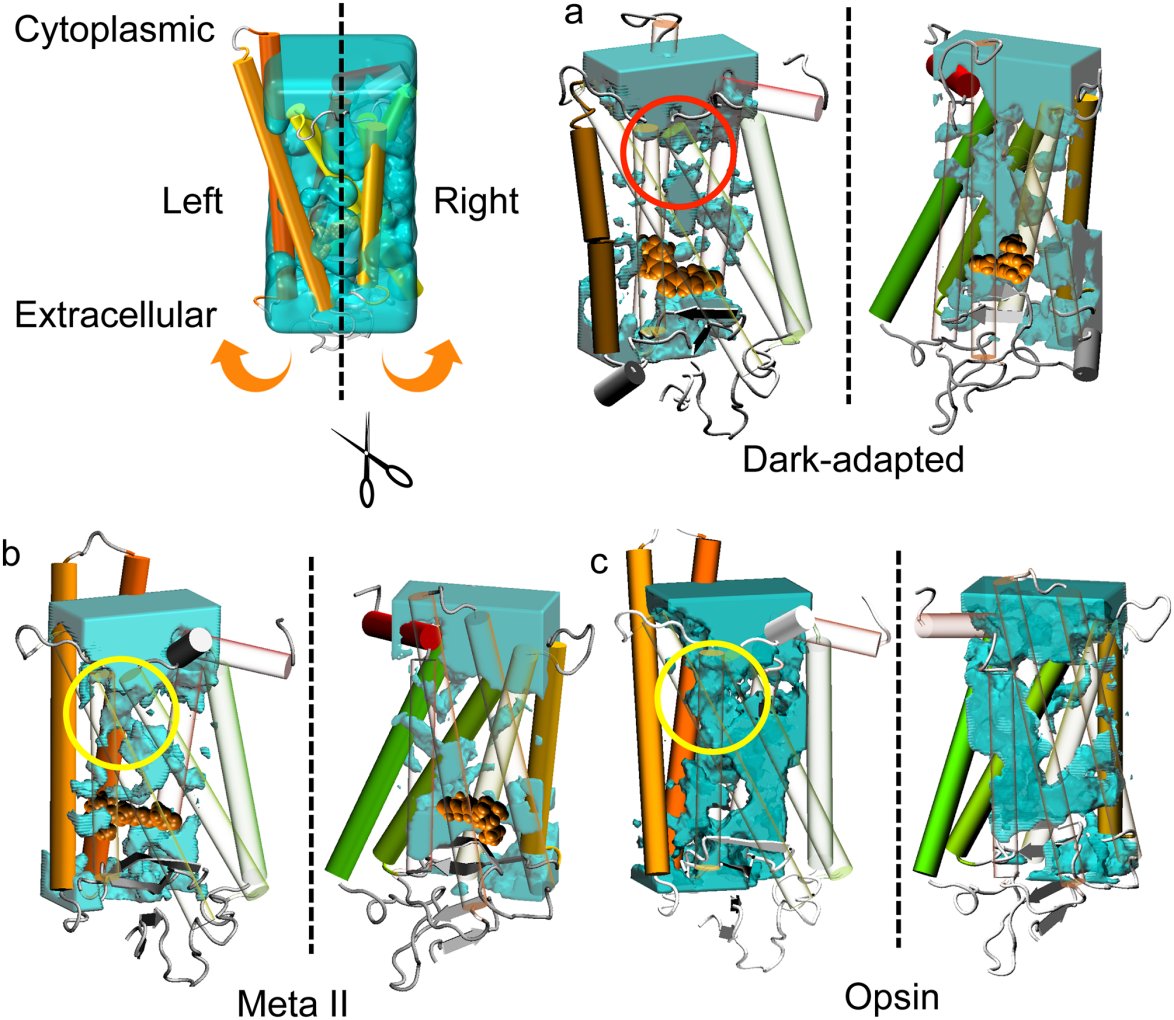
Accessibility of water molecules. Cross sectional diagrams of water accessibility with the cross section taken at the middle of the rhodopsin in (a) the dark-adapted rhodopsin, (b) the Meta II state, and (c) the Opsin. Blue surfaces represent places where water molecules have reached during last 0.8 *μ*s in the equilibrium state (see Fig 1f). The protein molecules are depicted with each helix colored from red (the N terminus) to green (the C terminus). The retinal is shown in orange VDW format. A solvent pore can be identified in the Meta II state and the Opsin (yellow circle). However, the solvent pore does not exist in the dark-adapted rhodopsin (red circle).

Rhodopsins have many internal water molecules and a flux of water molecules in its inside. Fig 2 shows regions where water molecules have accessed during the simulation. A putative solvent pore was identified on the cytoplasmic side in the Meta II state and the Opsin (see Fig 2b and 2c). This solvent pore flows from the cytoplasmic bulk to the retinal-binding pocket. However, in the dark-adapted rhodopsin the solvent pore is clearly separated at the entrance of the solvent pore (see Fig 2a). These results show that the solvent pore emerges after Meta II state due to the conformational change of the secondary structure. To validate the reproducibility of these results, we also performed MD simulations for three states of the rhodopsin using a different force field. Notably, the solvent pore was also observed in the Meta II state and the Opsin, while the solvent pore does not exist in the dark-adapted rhodopsin (see S1 Fig).

In all states, the solvent pore has hourglass-shaped entrance at the cytoplasmic site (see S2 Fig). When the water molecules enter into the rhodopsin from the cytoplasmic side, they pass through the hourglass-shaped entrance, which mediates optimal water permeation in the case of aquaporin water channels [44]. These results show that amino acids around the entrance become closed in the dark-adapted rhodopsin. Although the radius of the solvent pore is wider than the entrance of aquaporin [45], water molecules are not able to easily pass through the solvent pore. This is attributed to two narrow regions shown in the following analyses.

### Configuration of the solvent pore in the Meta II state

**Fig 3:**
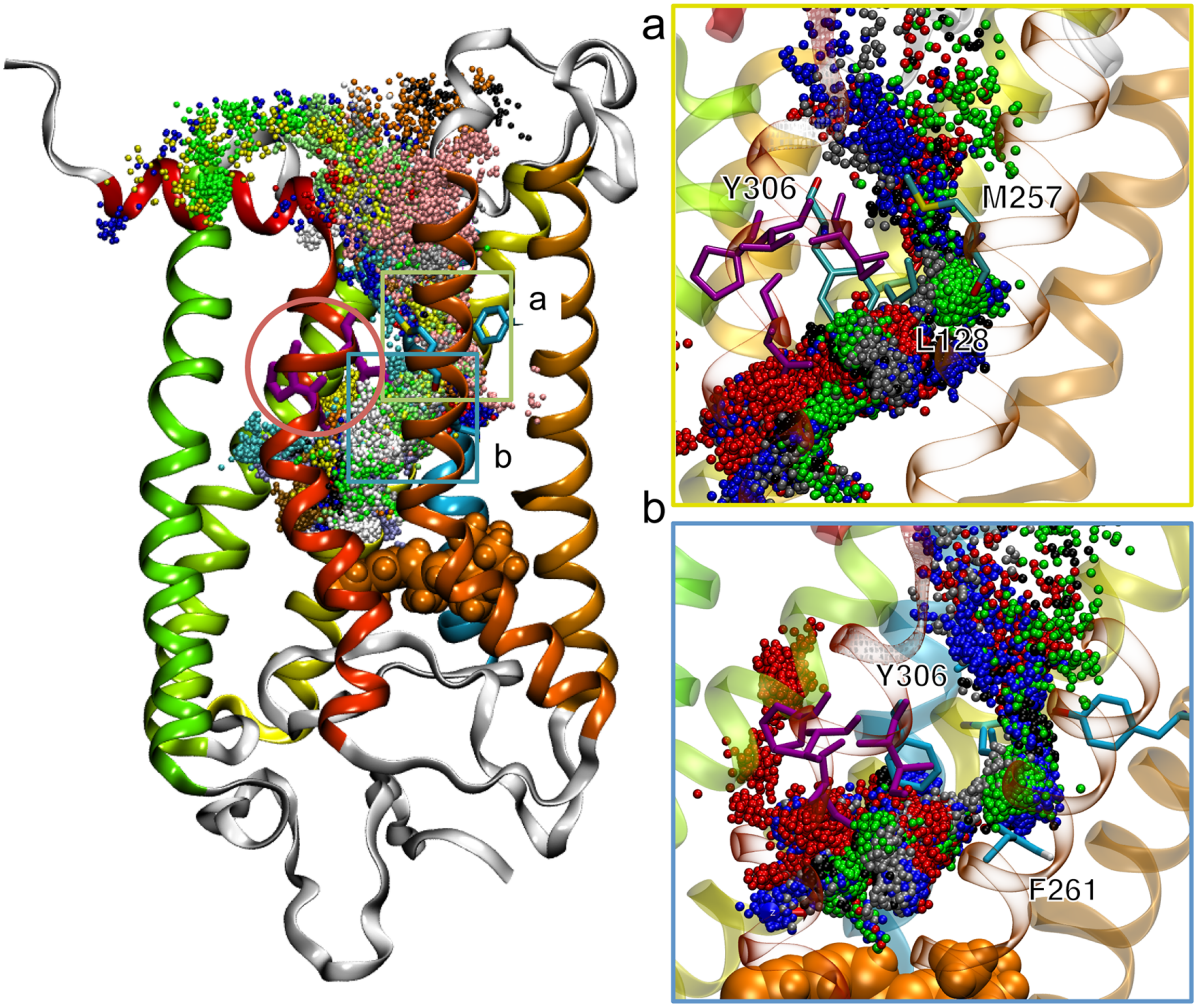
Configuration of the solvent pore on the cytoplasmic side with ribbon representation of the Meta II structure. Extracted 16 trajectories of water molecules that passed through the solvent pore are shown in different colors. Residues of the NPxxY are shown as purple and in the red circle. The retinal is shown in orange VDW format. (a) Close-up view of the first narrow region and (b) the second region are shown. Only five trajectories are shown. The first narrow region comprises L128, M257, and Y306, and the second narrow region comprises F261 and Y306.

Water molecules pass bi-directionally through the solvent pore. From the 1 *μ*s MD simulation of Meta II state, we could observe 16 times permeations of water molecules through the solvent pore between the bulk and the retinal-binding pocket. All the water molecules reached the K296, which means that the solvent pore can provide water molecules from the bulk to the Schiff base linkage. Fig 3 shows extracted trajectories of 16 water molecules passing through the solvent pore. The trajectories reveal two narrow regions that distinguish the bulk and the internal hydration sites. The first narrow region, which is bent into the L shape (see Fig 3a), comprises three amino acids, L128, M257, and Y306. The water pathway is narrow owing to the L128 and M257 amino acids, which are parallel to *z* axis. Below this region, the water pathway becomes bent due to Y306, and it is connected to internal hydration sites. The second narrow region comprises two amino acids, F261 and Y306. The two narrow regions are hydrophobic due to three amino acids, leucine, tyrosine, and phenylalanine. We calculated the distance distributions of the amino acids, and the presence of single peak indicates that the solvent pore have only one state (see S3a Fig).

After passing through the two regions, water molecules flow into important hydration sites, including the sites adjacent to the conserved structural motif, which is called “NPxxY”. This NPxxY motif, which is highly conserved in the GPCR family, is crucial for activation of GPCRs, and comprises asparagine (N; 75%, D; 21%), proline (P; 96%), two hydrophobic residues X and tyrosine (Y; 92%, F; 3%) [46]. The internal water molecules adjacent to the NPxxY motif mediate GPCR activation and stabilize the active state of GPCRs [26, 47].

These results mean that the solvent pore provides water molecules not only for the retinal-binding pocket, but also for internal hydration sites adjacent to the key conserved motif NPxxY.

### Water displacements within the solvent pore in the Meta II state

**Fig 4:**
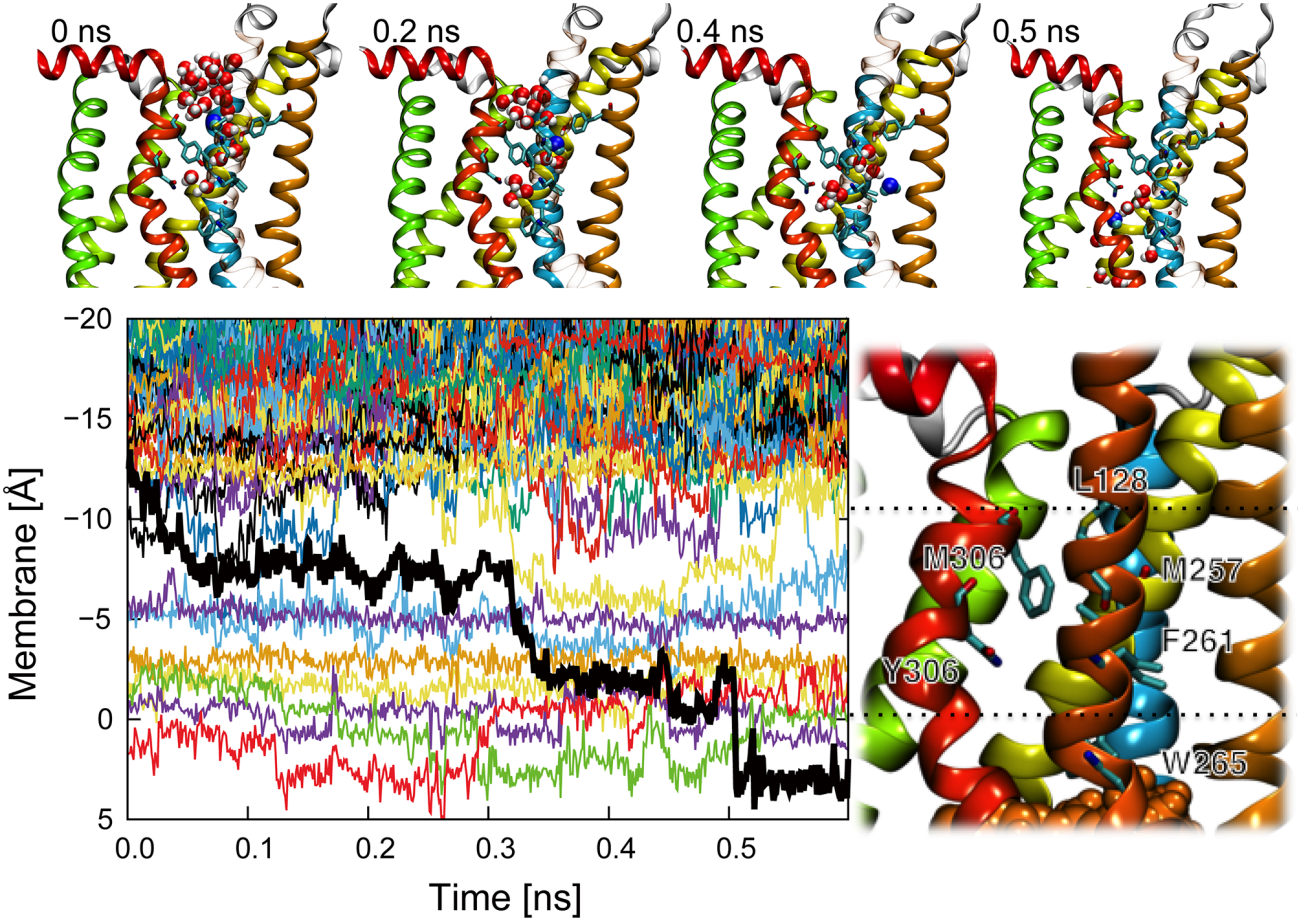
Water displacements in *z*-coordinate within the solvent pore when one water molecule passes through the solvent pore. The trajectory of the target water molecule is shown by a thick black line. The hydrophobic region and some internal hydration sites (from −10 to 0 Å) correspond to the area between the first and second regions. The trajectory has four characteristic states and upper snapshots express each characteristic state with the target water colored as blue. Residues of two narrow gates are shown as cyan. Positions 1, 2 and 3 are located between −10 and −5 Å, −5 and 0 Å, and 0 and 5 Å, respectively. Other 15 water displacements observed in the 1 *μ*s MD simulation are shown in S5 Fig.

**Fig 5:**
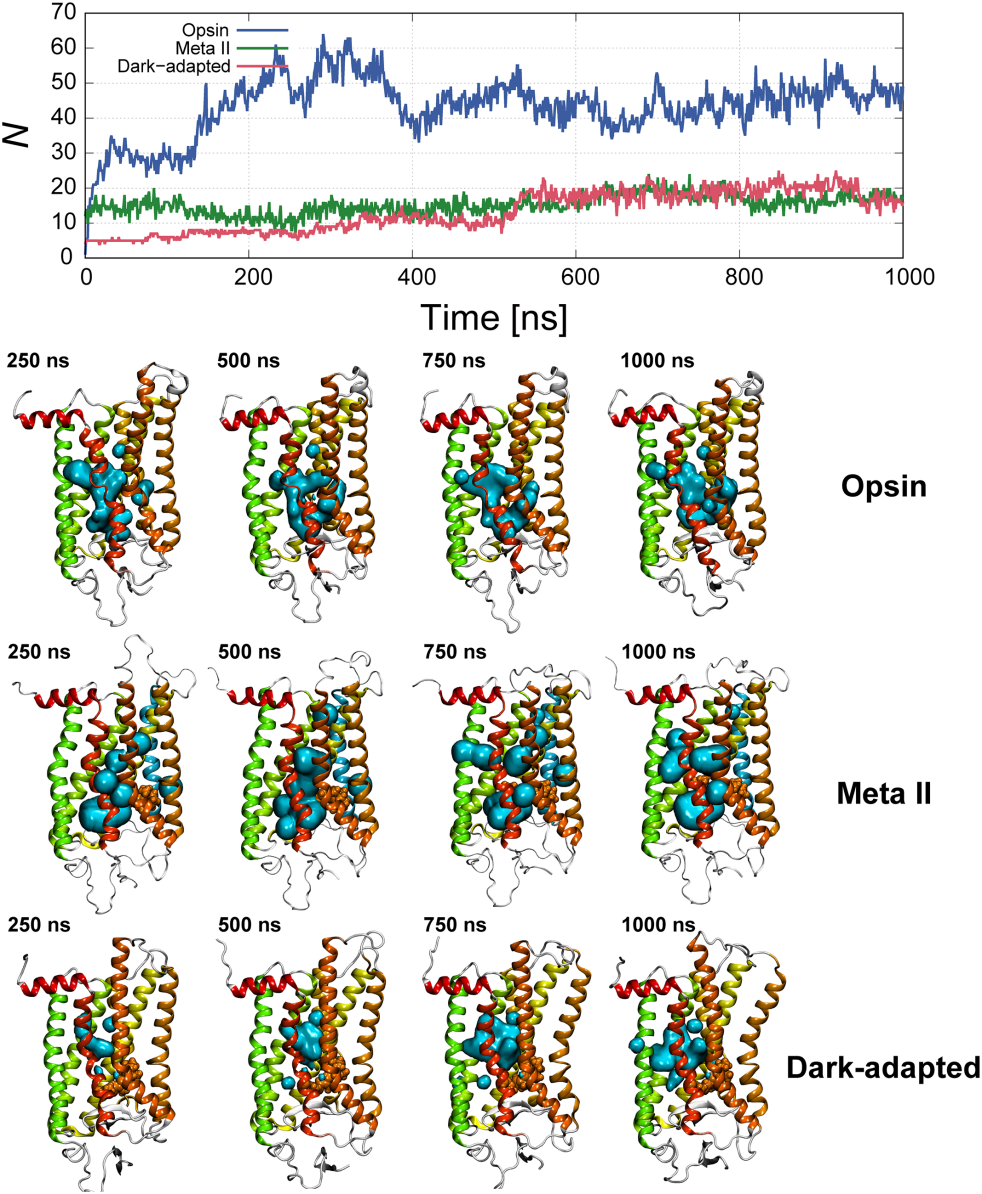
Times series of the number of internal water molecules in each state. The distributions of the internal water molecules (transparent cyan surface) are shown in the lower figures. The definition of internal water molecules is that water molecules within 7 Å of some amino acids located on the center of helixes.

The analysis of water trajectories in the solvent pore provides more details about the permeation process of water molecules [48]. Fig 4 shows water displacements in *z*-coordinate within the solvent pore when one water molecule passed through the solvent pore (other 15 trajectories of water permeation observed during 1 *μ*s MD simulation are shown in S4 Fig). There are three characteristic positions in the trajectories distinguished by the two narrow regions. The entrance of the solvent pore (less than −10 Å) is filled with bulk water, and most of the water molecules are blocked by the first narrow region which is composed of L128, M257 and Y306. The first narrow region is located between the entrance and the nearest internal hydration site (position 1). After this position 1, there is the second narrow region which is composed of F261 and Y306. The second narrow region is located between the positions 1 and 2. Internal hydration sites between the position 2 and sites around the retinal (position 3) are separated by N302 and W265. We found stable hydration sites (around −5 and −2.5 Å) in positions 1 and 2 (see S3b Fig). The former water molecule has a hydrogen bond with Y306 and M257 as a donor, while the latter water molecule has a hydrogen bond with Y306 as an acceptor. Other water molecules that pass through the solvent pore are also trapped in these positions (see S4 Fig). Because the two hydration sites are stable, we could always identify water molecules within the two hydration sites during the simulation. These water molecules have been already reported as the extended hydrogen bond network using the crystal structure [14]. Time series of the number of internal water molecules show that these internal water molecules were stable during our simulations, in particular in the Meta II state (see Fig 5). The definition of internal water molecules was that water molecules within 7 Å of some amino acids located on the center of helixes, where G51, V87, F88, I123, I217, F261, V300, Y301, and N302 are chosen for the dark-adapted rhodopsin; and G51, I123, A124, L125, W126, S127, I217, and K296 are chosen for the Meta II state and Opsin. We note that the number of internal water molecules in MD simulations is generally more than that in the crystal structure, because MD simulations can consider all the internal water molecules which include dynamical water molecules. When a water molecule passes through the narrow regions, the water molecule jumps or replaces a preceding water molecule. These results show that unlike in aquaporin, where water molecules diffuse in single file, water molecules in the solvent pore diffuse by pushing or sometimes jumping a preceding water molecule due to the geometry of the solvent pore.

### All water pathways between the bulk and the retinal-binding pocket in the Meta II state

As we mentioned above, the direction of water molecules through the solvent pore was not unidirectional. Therefore, one or more other pathways are needed for the equilibration of internal water molecules. The accessibility of water molecules (see Fig 1) also indicated the presence of other pathways. Extracted trajectories of water molecules passing between the bulk and the retinal-binding pocket allowed us to observe other pathways. First, we found that a cleft between TM4 and TM5 also becomes functional as a water pathway (see S5 Fig). Results from a previous study using random acceleration MD simulation suggested that the biggest cleft exists between TM4 and TM5 [11]. We also observed that water molecules passed through the two ligand pores. The ligand pore between TM1 and TM7 is composed of M39, L40, Y43, M44, F91, F94, T289, and F293, and the ligand pore between TM5 and TM6 is composed of V204, I205, M207, F208, A272, F273, and F276 [13]. Two ligand pores also become functional as water pathways in the present study. A water molecule penetrated into the membrane from the cytoplasmic side after diffusing on the membrane. Since the insides of the two ligand pores are hydrophobic, the number of water molecules which passed through the ligand pores is few. There are two mechanisms for water permeation across the membrane. (i) Although membranes in our systems are stable (see S6 Fig), the water molecule penetrates into the membrane. Then, since the inside of the membrane is high free energy for water molecules [49], the water molecule evacuates the inside of the membrane, which consequently leads to entrance into the cleft. (ii) The water molecule reaches the cleft along the interface between the protein surface and the membrane.

## Discussion

Overall, using all-atom MD simulations, we have examined the identification of the putative solvent pore on the cytoplasmic side. The solvent pore was identified in the Meta II state and Opsin, which is consistent with the experimental results. In the solvent pore, there are two narrow regions that distinguish the bulk and the internal hydration sites. The solvent pore also passes through the important hydration sites adjacent to the conserved structural motif “NPxxY”. We also revealed that unlike in aquaporin, where water molecules diffuse in single file, water molecules in the solvent pore diffuse by pushing or sometimes jumping a preceding water molecule due to the geometry of the solvent pore.

In association with conformational changes, the number of internal water molecules also changes through the intermediate states [50, 51]. A previous study showed that internal water molecules contribute to the thermodynamic stability of the entire rhodopsin [26]. In particular, the internal water molecules around the retinal stabilize local electric fields by hydrating near polar and charged amino acids [19]. In this study, we also identified the stable internal water molecules around the retinal, which has been reported in crystal structure [30]. These water molecules have hydrogen bonds with E113, E181, S186, K296 and the retinal. It was also suggested that internal water molecules contribute to functional plasticity and mediate the structural transitions from the dark-adapted rhodopsin to Opsin in all family A GPCRs [16].

Ronny *et al.* suggested that the solvent pore mediates water access [13]. We were able to show that the flow in solvent pore connects to the retinal-binding pocket, and the solvent pore also passes across the important hydration sites, such as the sites adjacent to the NPxxY motif and the extended hydrogen bond network. These results suggest two significance of the solvent pore: (1) the solvent pore provides water molecules to the retinal-binding pocket, and (2) the configuration and amount of important internal water molecules are controlled through the solvent pore.

## Supporting information

S1 Fig Accessibility of water molecules. Cross sectional diagrams of water accessibility with the cross section taken at the middle of the rhodopsin in (a) the dark-adapted rhodopsin, (b) the Meta II state, and (c) the Opsin using Amber force field. Blue surfaces represent places where water molecules have reached during the last 0.2 *μ*s. The protein molecules are depicted with each helix colored from red (the N terminus) to green (the C terminus). The retinal is shown in orange VDW format. A solvent pore can be identified in the Meta II state and the Opsin (yellow circle). However, the solvent pore does not exist in the dark-adapted rhodopsin (red circle).
(TIF)

S2 Fig Hourglass-shaped entrance of the solvent pore at the cytoplasmic site. Right figure shows the channel radius versus position along the pore axis. The center of the membrane is at 0 Å. The scale of the membrane corresponds with the left figure. The error bars drawn as transparent are given by standard deviation. The solvent pore is shown in Fig 2.
(TIF)

S3 Fig Characteristic regions within the solvent pore. (a) Distance distribution of amino acids related to the narrow regions. The distance was calculated between the nearest two atoms. The first narrow region is composed of L128, M257 and Y306, and the second narrow region is composed of F261 and Y306. The distance distributions of amino acids related to the narrow regions are unimodal. (b) In hydrophobic layer, two stable hydration sites are stable during the simulation. Red water molecule has hydrogen bond with Y306 and M257 as donor (z coordinate is -5 Å). Orange water molecule has hydrogen bond with Y306 as acceptor (z coordinate is -2.5 Å).
(TIF)

S4 Fig Trajectories of all water molecules which passed through the solvent pore in z coordinate. The positions 1 and 2 are separated by the first narrow region which is composed of L128, M257 and Y306. The positions 2 and 3 are separated by the second narrow region which is composed of F261 and Y306.
(TIF)

S5 Fig Other water pathways between the bulk and the retinal-binding pocket. Blue spheres show trajectories of oxygen atoms in a water molecule every 1 ps. (a) Water pathway through the cleft between TM4 and TM5. Each color shows trajectories of different water molecules. (b) and (c) show trajectories of water molecules through the ligand pore A and ligand pore B, respectively.
(TIF)

S6 Fig Stability of the simulation systems. (a) Area (box size Lx×Ly) and (b) thickness (difference between averaged z coordinates of phosphorus atoms in each leaflet) of membrane in each simulation.
(TIF)

S1 Video Trajectory of the water molecule passing through the solvent pore. (MP4)
