# Supplementary figures and images for: Water permeation through the internal water pathway in activated GPCR rhodopsin
Source: PLoS One. 2017 May 11;12(5):e0176876. doi: 10.1371/journal.pone.0176876 (PMC5426653; doi:10.1371/journal.pone.0176876)

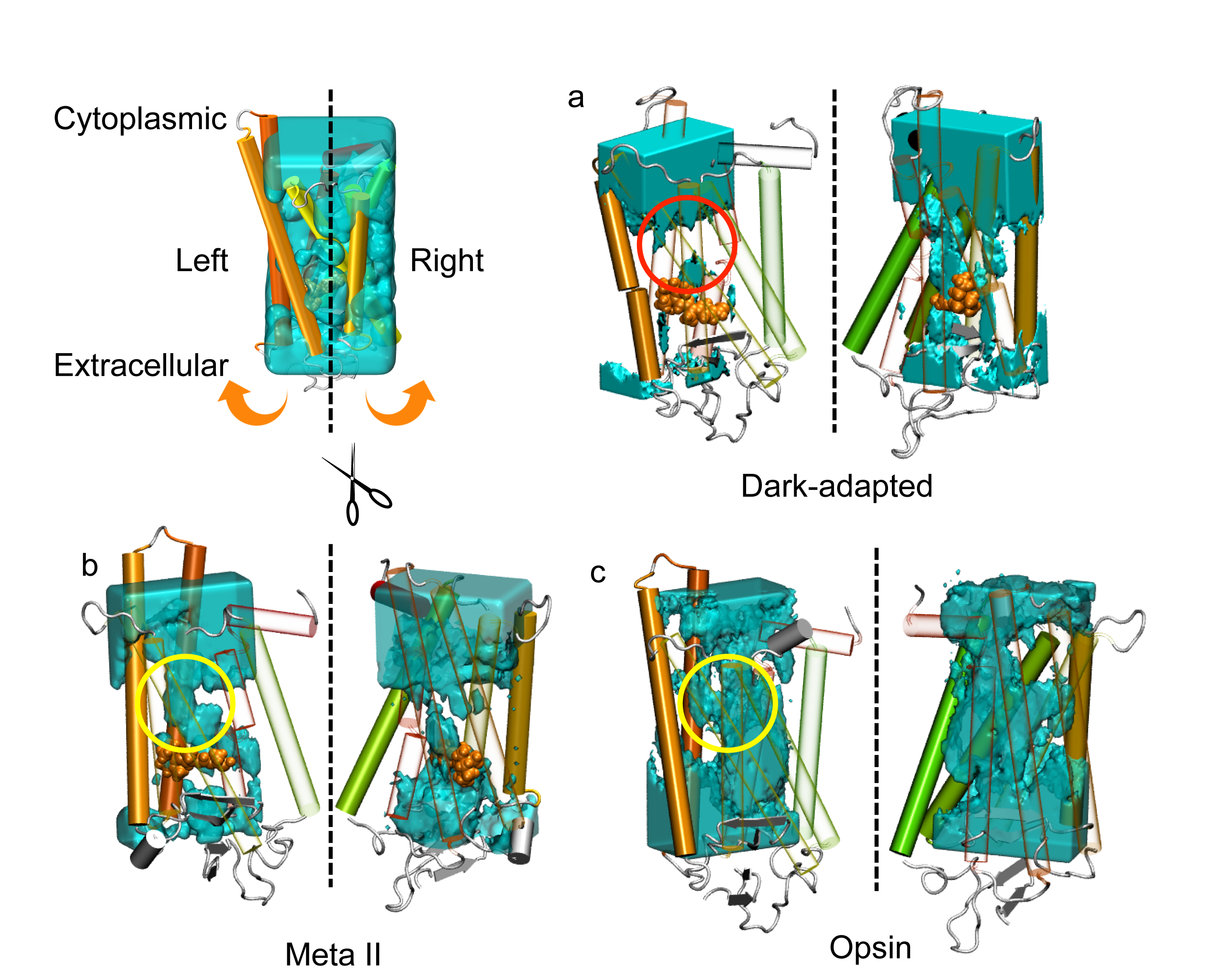

Supplement: S1 Fig — Cross sectional diagrams of water accessibility with the cross section taken at the middle of the rhodopsin in (a) the dark-adapted rhodopsin, (b) the Meta II state, and (c) the Opsin using Amber force field. Blue surfaces represent places where water molecules have reached during the last 0.2 μs. The protein molecules are depicted with each helix colored from red (the N terminus) to green (the C terminus). The retinal is shown in orange VDW format. A solvent pore can be identified in the Meta II state and the Opsin (yellow circle). However, the solvent pore does not exist in the dark-adapted rhodopsin (red circle). (TIF) [file pone.0176876.s001.tif]

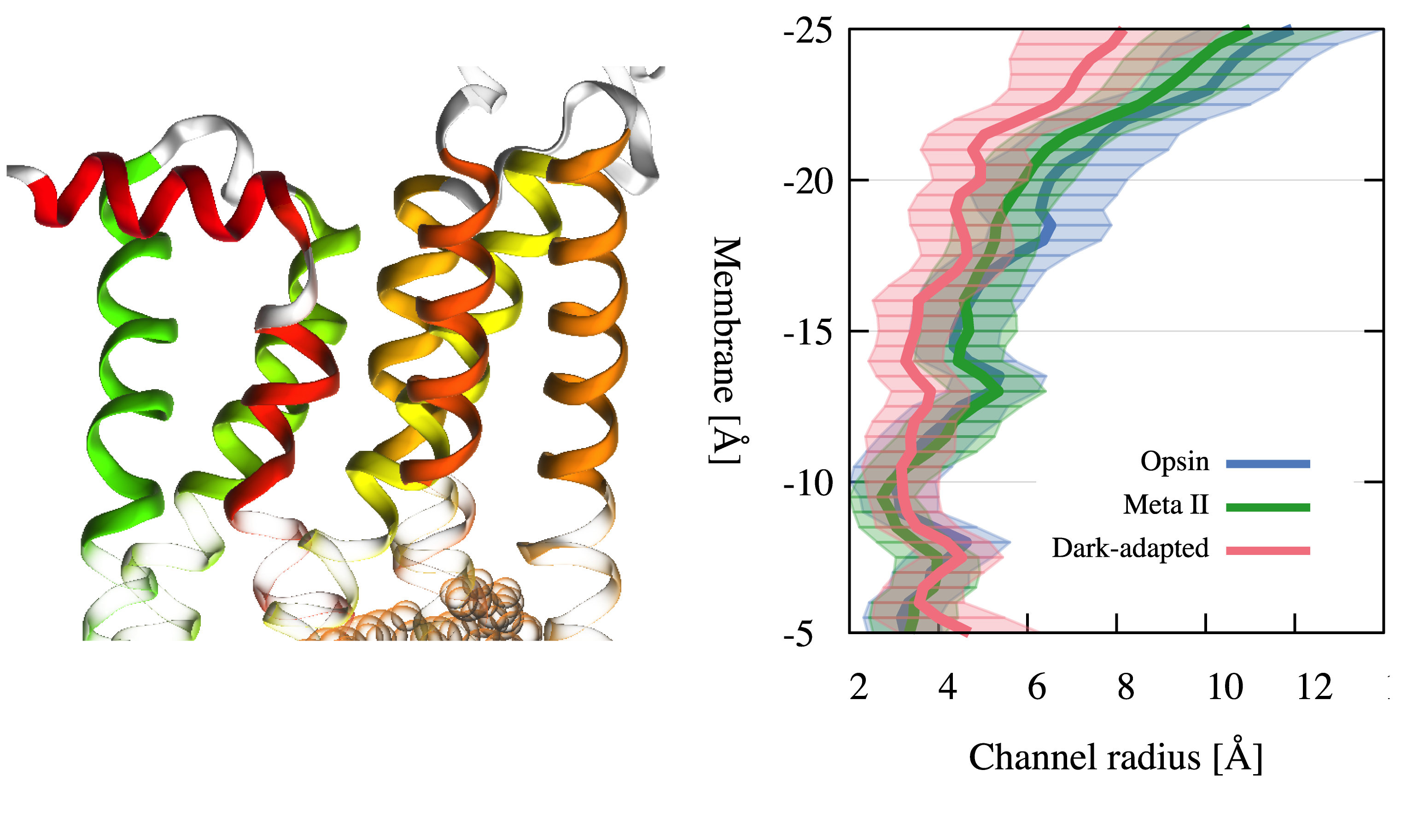

Supplement: S2 Fig — Right figure shows the channel radius versus position along the pore axis. The center of the membrane is at 0 Å. The scale of the membrane corresponds with the left figure. The error bars drawn as transparent are given by standard deviation. The solvent pore is shown in Fig 2. (TIF) [file pone.0176876.s002.tif]

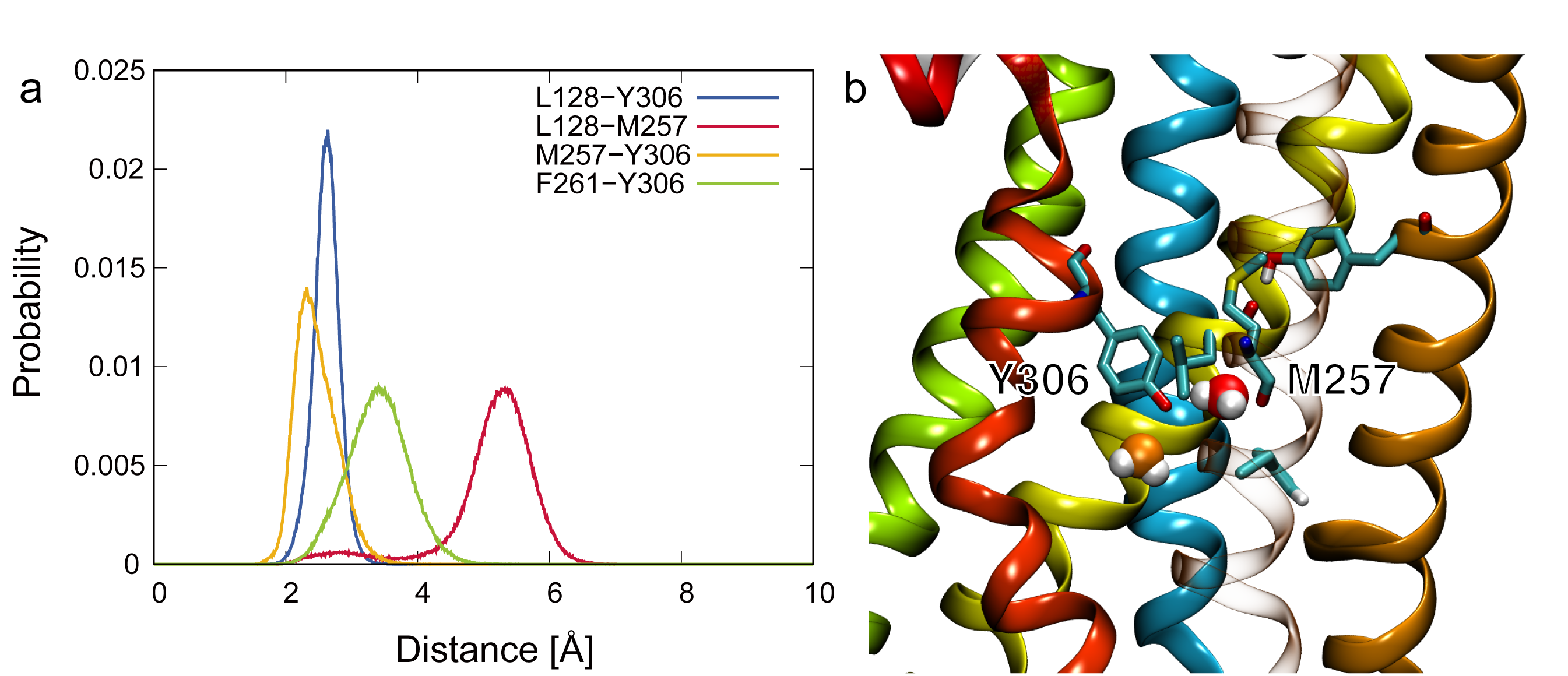

Supplement: S3 Fig — (a) Distance distribution of amino acids related to the narrow regions. The distance was calculated between the nearest two atoms. The first narrow region is composed of L128, M257 and Y306, and the second narrow region is composed of F261 and Y306. The distance distributions of amino acids related to the narrow regions are unimodal. (b) In hydrophobic layer, two stable hydration sites are stable during the simulation. Red water molecule has hydrogen bond with Y306 and M257 as donor (z coordinate is -5 Å). Orange water molecule has hydrogen bond with Y306 as acceptor (z coordinate is -2.5 Å). (TIF) [file pone.0176876.s003.tif]

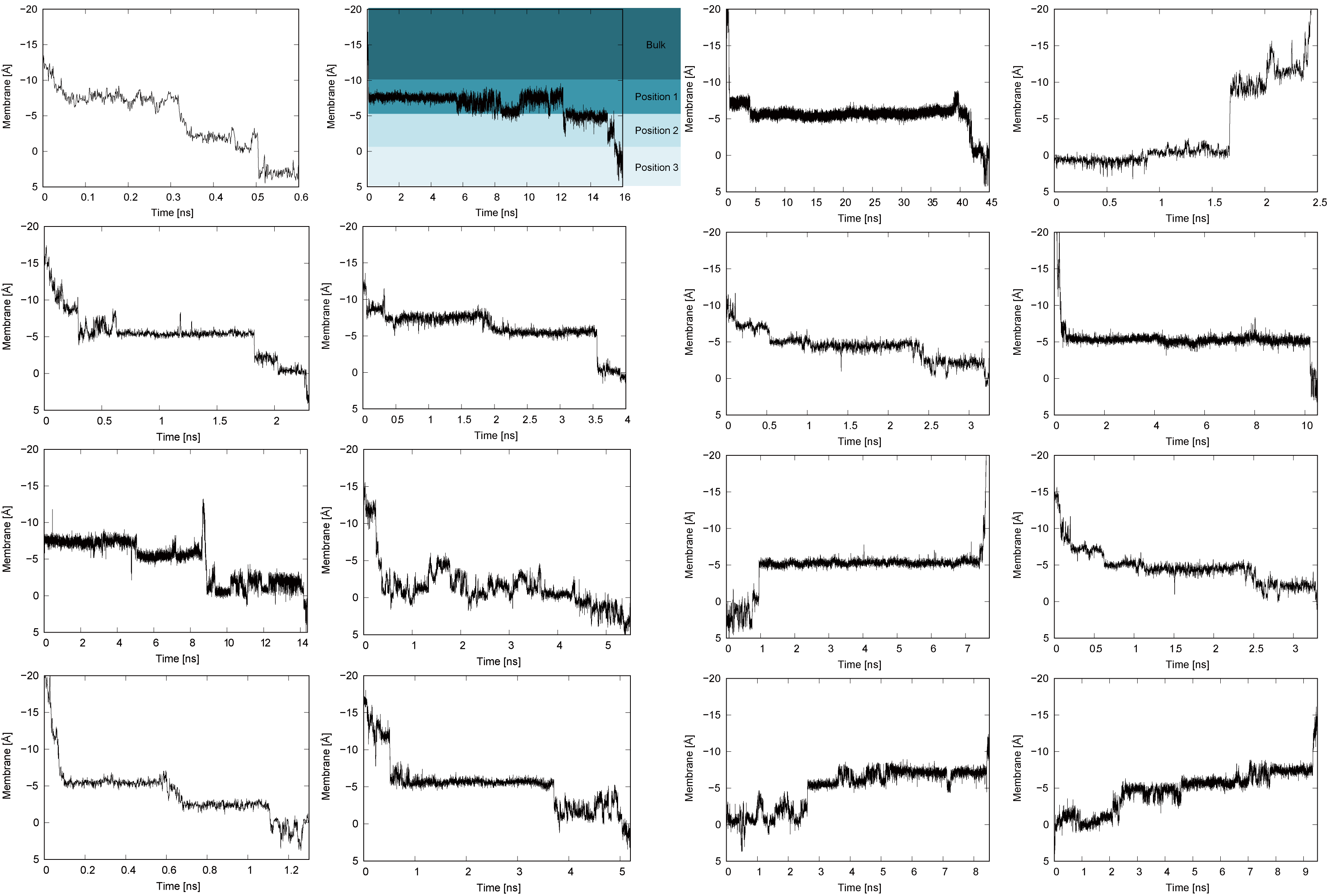

Supplement: S4 Fig — The positions 1 and 2 are separated by the first narrow region which is composed of L128, M257 and Y306. The positions 2 and 3 are separated by the second narrow region which is composed of F261 and Y306. (TIF) [file pone.0176876.s004.tif]

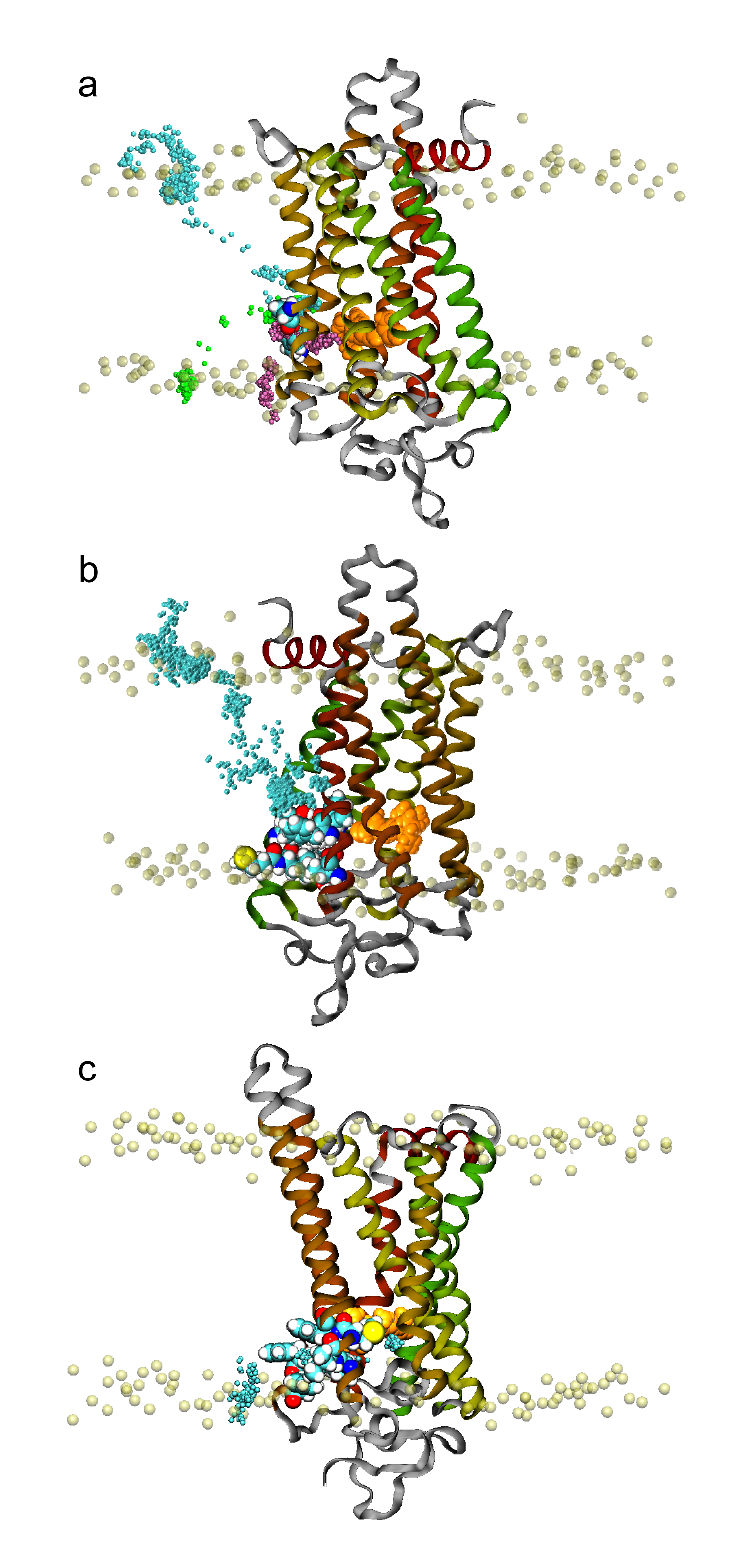

Supplement: S5 Fig — Blue spheres show trajectories of oxygen atoms in a water molecule every 1 ps. (a) Water pathway through the cleft between TM4 and TM5. Each color shows trajectories of different water molecules. (b) and (c) show trajectories of water molecules through the ligand pore A and ligand pore B, respectively. (TIF) [file pone.0176876.s005.tif]

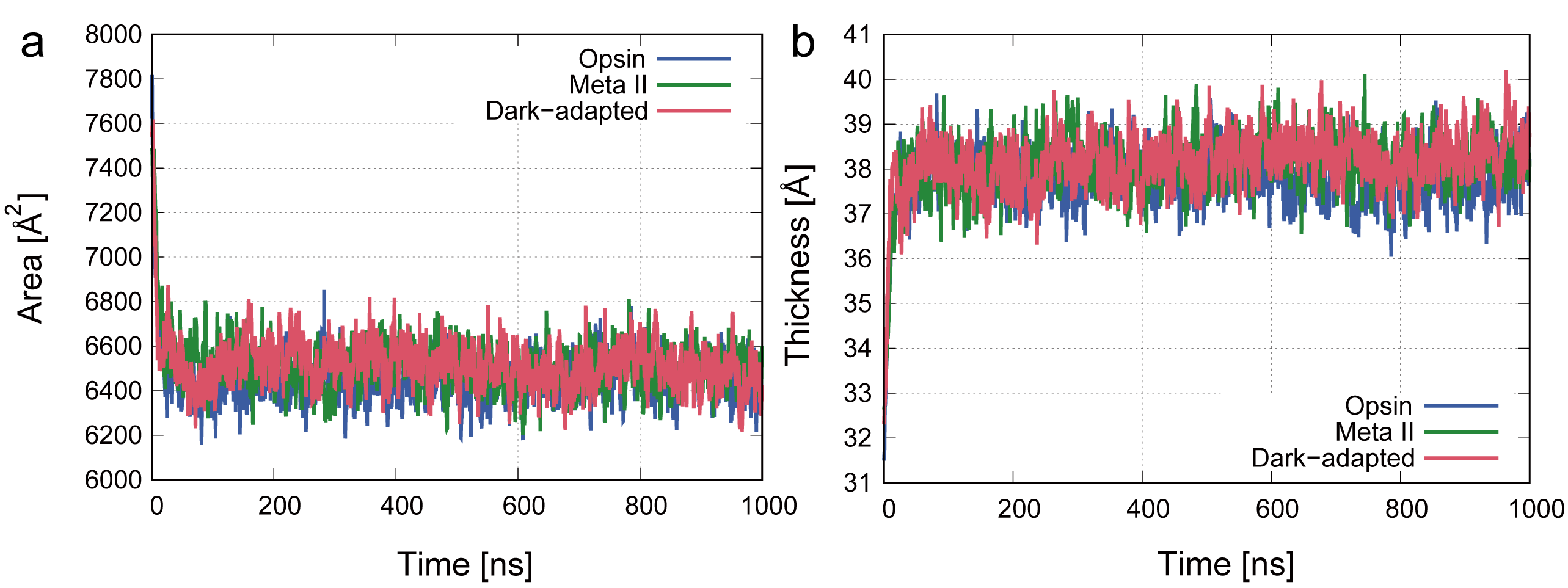

Supplement: S6 Fig — (a) Area (box size Lx×Ly) and (b) thickness (difference between averaged z coordinates of phosphorus atoms in each leaflet) of membrane in each simulation. (TIF) [file pone.0176876.s006.tif]
